# Simple reaction time and obesity in children: whether there is a relationship?

**DOI:** 10.1186/s12199-017-0612-0

**Published:** 2017-03-14

**Authors:** Akbar Moradi, Samad Esmaeilzadeh

**Affiliations:** 1 University of Mohaghegh Ardabili, 153 Nasim 1, part III of Sabalan, 5619888457 Ardabil, Iran; 2 Department of Education, Ardabil, Iran; 3 Islamic Azad University science and research Branch, Tehran, Iran

**Keywords:** Audio-visual reaction time, Body mass index, Clinical reaction time, Cognitive function, Fat percentage, Waist circumference, Waist to height ratio

## Abstract

**Objective:**

Reaction time (RT) testing is one of the oldest diagnostic methods used in modern psychology, and is known as simple and sensitive cognitive test. It has been recently reported that RT is related to obesity in young, adult and elderly individuals. However, most of the studies included small sample of participants, used just body mass index (BMI) as body obesity index, and did not consider some potential confounders such as age, socioeconomic status and physical activity in their studies. Furthermore, there is little and contradictory results for children. Therefore, the present study aimed to examine the relationship between RT and weight status in a sample of children.

**Methods:**

Three hundred and fifty four 9–12 year old schoolboys underwent standard anthropometry, and various simple RT tests.

**Results:**

After controlling for potential confounders no significant relationship was observed between audio-RT (RTA) and clinical RT (RT_clin_) with BMI, %fat, waist circumference (WC) and waist to height ratio (WHtR) (*P* > 0.05). But, significant relationship (β = 0.18; *P* = 0.02) was observed between visual-RT (RTV) and %fat (but not BMI, WC and WHtR).

**Conclusions:**

Among the various simple RT tasks and central and total body obesity indices, just significant relationship was observed between %fat and RTV in the schoolboys. According to the results, it is concluded that RT impairment due to obesity may less be observed, or may not be observed for some types of RT tasks and obesity indices during childhood.

## Introduction

Reaction time (RT) testing is one of the oldest diagnostic methods used in modern psychology, and the first examinations of this parameter dates back to the nineteenth century [1]. RT tests are known as simple and sensitive cognitive test in both healthy individuals and patients [2]. It is the time interval between the using of a stimulus and the appearance of quick voluntary response by an individual. It involves three phases as follows: a) processing of the stimulus, b) making decision about it and c) programming a response. Therefore, RT measurement includes: 1- The sensory neural code latency traversing both in central and peripheral pathways, 2- Both cognitive and Perceptive processing, 3- A motor signal traversing both peripheral and central neuronal structures 4- And eventually the latency in the end effectors activation such as muscle activation [3]. Speed of human’s information processing and its quality can be evaluated by using one (simple RT) or more (Choice RT) stimuli, and from an executive functions perspective both tasks are a measure of sustained attention; however, choice RT task put greater stress on the decision making and leads prolonged RT than simple RT [4].

It has been shown that some factors such as age [5–7], caffeine and some drugs [8, 9], illnesses [10], socioeconomic status (SES) [6, 7, 11], and physical activity (PA) lifestyle patterns [6, 7, 12, 13] are associated with RT. There is also some evidence has recently been reported by researches underlying relationship between obesity and RT in young, adult and elderly individuals [14–20]. The evidence suggests that overweight/obese individuals are inferior while performing RT compared with their healthy weight peers [14–20]. But, contradictory results underlying the relationship between obesity and RT have been reported for children [5, 6].

Regarding the relationship between obesity and RT that have been reported recently some points should be noted. For instance, most of the previous studies underlying the relationship between RT and obesity included small sample of participants and/or used just body mass index (BMI) as the obesity index [15–20], while BMI can be influenced by a number of factors and indices, such as central obesity, which is more closely linked to some adverse health outcomes than BMI [21], and therefore obesity indices may differentially relate to changes in cognitive function over time. In addition some previous similar studies did not consider some potential confounders such as age, socioeconomic status (SES) and PA lifestyle patterns [15–20], whereas it has been reported that SES is strongly correlated with cognitive ability and achievement during childhood and beyond [11]; and it has been shown that more physically active individuals are better able to respond quickly to a stimulus presented to [6, 7, 12] and are capable of allocating more attentional resources toward the environment and process information more quickly [13].

In this context the purpose of the present study was to examine whether there is significant relationship between various RT tasks and obesity indices among a relatively large numbers of children after controlling for some potential confounders such as age, SES and PA.

## Methods

### Participants

**Fig. 1:**
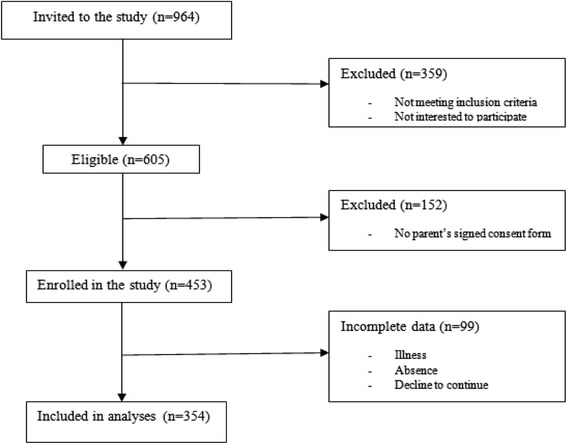
Flow diagram demonstrating the study methodology

During 2013–2014, a cross-sectional data were drawn from a sample of three hundred and fifty four, 9–12 year old schoolboys of three schools, in the center of Ardabil Province, North West of Iran. Three schools were selected randomly from a list of boys’ urban public schools (*N* = 130). The nature and purpose of the study were explained to all schoolboys (*n* = 964) before invitation for participating in the present study. Children were excluded of the study if they were identified with known presence of chronic disease or using any medication which could affect RT [9, 10]. Children who were invited and passed the exclusion criteria of the study and gave their consent verbally (*n* = 605) were given written consent forms for their parents approval. Total of four hundred fifty three signed consent forms were collected and the owners of the forms were recruited into the study. Nonetheless, at the end of the study, complete data were collected from 354 participants [dropped data because of illness, absence and withdrawal for some personal reasons etc.] (Fig. 1). Age of the boys was determined from their date of birth in their school register. The anthropometric variables and RT tests were measured in the empty room. The measurements took place when a participant was at rest. For this mean, during the physical education lessons selected boys were requested to undergo the measurements. All measurements were performed during the winter and spring of the year 2013. General characteristics of the participants are indicated in Table 1. The present study was approved by the Human Ethics Committee of the Ardabil Department of Education, and the experiment was performed in accordance with the ethical standards of the committee and with the Helsinki Declaration.

**Table 1:** General characteristics of the boys (*n* = 354)

	Mean	(SD)
Age (year)	10.90	(0.9)
Height (m)	1.40	(0.1)
Weight (kg)	42.00	(11.6)
%fat	25.20	(10.1)
WC (m)	0.68	(0.1)
WHtR	0.476	(0.1)
BMI (kg/m^2^)	20.10	(3.9)
PA (score)	2.70	(0.8)
RT_clin_ (s)	0.240	(0.08)
RTA (s)	0.350	(0.05)
RTV (s)	0.342	(0.06)

*BMI* body mass index, *PA* physical activity, *RT*_clin_ clinical reaction time, *RTA* audio reaction time, *RTV* visual reaction time, *WC* waist circumference, *WHtR* waist to height ratio

### Anthropometric variables and obesity indices

Height of the participants was measured barefoot in the Frankfurt horizontal plane with a telescopic height measuring instrument (Type SECA 225) to the nearest one mm. Weight of the participants was measured in without shoes and underwear with an electronic scale (Type SECA 861) to the nearest 0.1 kg.

BMI and %fat were measured as the total body obesity indices and waist circumference (WC) and waist to height ratio (WHtR) were measured as the central body obesity indices. BMI was calculated as body weight in kilograms divided by the square of height in meters. WC was measured midway between the superior border of the iliac crest and the lowest rib with an inelastic measuring tape at the end of normal expiration to the nearest 0.1 mm. WC was divided by the height to determine the waist to height ratio (WHtR). Lange skinfold caliper was used to assess triceps and calf skinfold thickness [22] on the right side of the body for all the subjects. The average of three measures was calculated for each site and then %fat was calculated according to the equations [22].

$$ \mathbf{Boys}\ \%\mathbf{fat} = 0.735\ \left( sum\  of\  average\  skinfolds\right) + 1.0 $$

### Reaction time (RT) tests

All participants were made familiar with procedure of the RT tests and were requested to avoid using caffeine containing drinks [8] and any acute PA [12]. The boys were made to sit comfortably in a chair and were motivated to better their results as much as possible, using the dominant hand, with a protocol of 5 practice trials for each RT tests followed by data acquisition trials. Data from practice trials were not included to analyses. In our experiments we used rest break times (5 min) between each RT test to prevent fatigue [23].

### Audio Reaction Time (RTA) and Visual Reaction time (RTV) tests

RT software which was installed on a laptop was used for obtaining both RTA and RTV which had an inter-stimulus interval from1000 to 3000ms, response range from 150 to 1500 ms, and a display accuracy of 0.001 s. By starting each RT test each subject completed 40 RTV and 40 RTA executions. For performing RTV the subject was requested to press a default key (spacebar) as soon as possible, using the index finger, which was in contact with the key, when a large dot (highlighting green circles against a yellow background) appeared on the monitor. For executing RTA the subject was requested to press the key every time he heard a “beep” sound. Headphone was provided for clarity of sound. After completing each test, average of performances for each test were recorded for each subject.

### Clinical Reaction Time (RT_clin_)

In addition to the above computerized simple RT tests we measured a new clinical measure of RT (RT_clin_) which has been recently developed and validated [24, 25]. Each boy participated in simple RT_clin_ testing, using the RT_clin_ apparatus, which has been described elsewhere [5–7, 24, 25] but is repeated here for convenience of the reader. The apparatus for measuring RT_clin_ is a measuring 0.8 m long stick, marked in 5 mm increments embedded in a weighted rubber disk. Each boy sat with the dominant forearm resting on a horizontal desk surface, such that the proximal edge of the hypothenar eminence was positioned at the edge of the desk. The apparatus was held by the examiner vertically, with the weighted disk put inside the boy’s open hand, such that the superior surface of the weighted disk was aligned with the plane of the boy’s first 2 digits and no part of his hand was in contact with the weighted disk. The apparatus was dropped by the examiner at predetermined, randomly assigned time intervals of between two and five seconds to prevent the boy from anticipating the time of drop. Then, the boy tried to catch the apparatus as quickly as possible after it began to drop. In the event of an anticipatory catch before the apparatus was dropped, the examiner restarted the random-delay interval count before dropping the apparatus. If the device was dropped by a boy that trial did not include in the calculation of mean RT_clin_. The boys were tested using the dominant hand with a protocol of 10 data acquisition trials. The distance the device fell before being caught by the boy was recorded in meters (m), and used to calculate RT_clin_ in seconds (s) for each trial using the formula for a body falling under the influence of gravity (t = 0.45 × √d); where “d” is distance (m) and “t” is time (s) [24, 25]. Mean baseline RT_clin_ values were calculated for each boy.

### Potential covariates/confounders

#### Physical activity (PA)

PA for the children was measured using the PA Questionnaire - Children (PAQ-C) a reliable and valid measure of PA for children during the school year [26–28] with some alternations to suit to our society [29, 30]. Children were requested to fill out the questionnaire under their parents’ supervision.

#### Socioeconomic status (SES)

SES was computed from parents’ education and occupational status which is explained previously [6, 7, 30, 31].

### Statistics

Descriptive statistics were run on all variables. Data were screened for problems of skew, kurtosis, and outliers. Initial Pearson product–moment correlations were conducted on relationship between RT tests and age, SES and PA score. Any variable exhibiting a significant correlation with the dependent variable (RT) was included as a covariate/confounder in the analyses. For finding the relationship between the RT tests and the obesity indices hierarchical regression analysis was conducted in 2 steps. In the first step confounding variables (age, SES and PA) were entered, and in the second step obesity indices were entered, separately. All calculations were performed using SPSS v.21.0 software for Windows (SPSS Inc, Chicago, IL, USA). The significance level was set at *p* < 0.05.

## Results

Pearson correlation (Table 2) indicated significant negative relationship between age and RTV, RTA and RT_clin_ (*P* < 0.01). Significant negative relationship was observed between SES and RTV, RTA and RT_clin_ (*P* < 0.01). Although, negative relationship was observed between PA and the RT tests, only significant relationship was observed between PA and RT_clin_ (*P* < 0.05).

**Table 2:** Pearson correlation between the RT tests and age, SES and PA

	RTV		RTA		RTclin	
	R	*P*	R	*P*	R	*P*
Age	−0.50	(*P* < 0.01)	−0.56	(*P* < 0.01)	−0.40	(*P* < 0.01)
SES	−0.22	(*P* < 0.01)	−0.35	(*P* < 0.01)	−0.30	(*P* < 0.01)
PA	−0.10	(*P* = 0.11)	−0.11	(*P* = 0.09)	−0.15	(*P* < 0.05)

*RT*_clin_ clinical reaction time, *RTA* audio reaction time, *RTV* visual reaction time, *PA* physical activity, *SES* socioeconomic status

Multiple linear regression analysis (Table 3) indicated significant negative relationship between age and the RT tests (*P* < 0.01). Significant negative relationship was observed between RT_clin_ and SES (*P* < 0.05). Significant negative relationship was found between PA and RT_clin_ and RTV (*P* > 0.05). Linear regression analysis after adjustment for the potential confounders (step 2) indicated significant relationship between RTV and %fat (*P* < 0.05). No significant relationship was observed between RTV, BMI, WHtR and WC (*P* > 0.05). In addition regression analysis indicated no significant relationship between RTA and RT_clin_ with the all obesity indices (*P* > 0.05).

**Table 3:** Relationship between the RT tests with possible confounders (step 1) and the obesity indices (step 2)

		RTclin	RTA	RTV
		β (*P*)	β (*P*)	β (*P*)
	Age	−0.29 (*P* < 0.01)	−0.54 (*P* < 0.01)	−0.52 (*P* < 0.01)
Step 1	SES	−0.17 (*P* = 0.02)	−0.03 (*P* = 0.63)	0.04 (*P* = 0.54)
	PA	−0.18 (*P* = 0.01)	−0.09 (*P* = 0.13)	−0.12 (*P* = 0.04)
	BMI	−0.03 (*P* = 0.69)	0.04 (*P* = 0.55)	0.13 (*P* = 0.07)
Step 2	%fat	−0.09 (*P* = 0.29)	0.03 (*P* = 0.70)	0.18 (*P* = 0.02)
	WC	−0.05 (*P* = 0.50)	−0.01 (*P* = 0.09)	0.06 (*P* = 0.41)
	WHtR	−0.08 (*P* = 0.29)	0.03 (*P* = 0.61)	0.07 (*P* = 0.30)

*BMI* body mass index, *PA* physical activity, *RT*_clin_ clinical reaction time, *RTA* audio reaction time, *RTV* visual reaction time, *SES* socioeconomic status, *WC* waist circumference, *WHtR* waist to hip ratio

Note: In the first step confounding variables (age, SES and PA) were entered, and in the second step obesity indices were entered, separately

## Discussion

The results indicated that among the various obesity indices and simple RT tests, and after controlling for the possible confounders only significant relationship was observed between RTV and %fat. No significant relationship was observed between RTV, BMI and central obesity indices; and no significant relationship was observed between RT_clin_ and RTA to the obesity indices.

In the previous studies among schoolboys no significant relationship was observed between RT_clin_ and various obesity indices (BMI, %fat, WC and WHtR), which the present study results replicate the previous study results [5, 6]. It has been suggested that type of the RT task might play an important role in the relationship between weight status and RT [5–7]. For instance, some recent studies reported contradictory results based on the relationship between type of task performance and obesity, and discrepancy results have been shown between healthy weight and obese children for different types of RT, and it has been concluded that “*childhood obesity is negatively and selectively associated with prefrontal inhibitory control*” [14, 32].

Although, several recent studies have reported positive relationship between RT and obesity (measured by BMI) among young, adult and older people [15–20], however, we observed that among a relatively larger sample of participants, using various obesity indices and RT tests and controlling for potential confounders just RTV was significantly related to %fat. In this context it should be stated that subcutaneous fat percentage as an overall body obesity index has been reported as a better body obesity predictor than BMI and has been concluded that BMI as a measure of total body obesity does not account for varying proportions of fat, muscle mass, and bone or the distribution of body fat [33, 34]. However, to the best of our knowledge little studies underlying the relationship between obesity and RT have used subcutaneous fat percentage as well as central obesity.

Interestingly, we observed negative (but not significant) relationship between RT_clin_ and obesity in the children. Our results are according to some recent studies that have reported it is not necessarily appropriate to consider body adiposity as a negative factor influencing neuromuscular RT performance, and in contrast body lipid reserves are integral to the development of the nervous system (e.g., the development of myelin nerve heath) and thus better RT, even amongst individuals within the healthy weight ranges [35]. Therefore, according to the results of the present study and literature [5–7, 17, 32, 35, 36] it is plausible that RT impairment due to obesity lees be observed during childhood, or may not be observed for some types of RT tasks and obesity indices.

However, since the study has several limitations, results need to be interpreted with caution. For example, the cross-sectional nature of the study limits the possibility to draw conclusions regarding causality of any of the observed associations in the present study. Furthermore, the present study did not include subjects of both sexes. Therefore, for more reliable results about the relationship between RT and weight status, additional studies by using larger sample size, various age groups, RT tests, obesity indices and in both sexes are needed.

## Abbreviations

BMI: Body mass index
PA: Physical activity
RT: Reaction time
RTA: Audio reaction time
RT_clin_: Clinical reaction time
RTV: Visual reaction time
SES: Socioeconomic status
WC: Waist circumference
WHtR: Waist to height circumference
